# Dietary α-linolenic acid improves skin blood flow following cold exposure and endothelial function in healthy adults: a randomized, double-blind, placebo-controlled, parallel-group comparative trial

**DOI:** 10.3389/fnut.2026.1878148

**Published:** 2026-07-31

**Authors:** Nanaka Ando, Naohisa Nosaka, Mayu Hasegawa, Kazuhiko Kato

**Affiliations:** 1 The Nisshin OilliO Group, Ltd., Yokohama, Kanagawa, Japan; 2 Kato Clinic, Komae, Tokyo, Japan

**Keywords:** allergy, blood flow, cardiovascular disease, cold sensitivity, FMD (flow mediated dilation), vascular endothelial function, α-linolenic acid

## Abstract

**Introduction:**

Previous studies have suggested that α-linolenic acid (ALA) improves vascular function and alleviates allergic symptoms. However, its effects on cold sensitivity, associated with peripheral vascular function, remain poorly understood. Furthermore, no studies have reported ALA’s efficacy alone in healthy humans regarding vascular endothelial function or allergic symptoms. This study aimed to clarify ALA intake effects on cold sensitivity, vascular endothelial function, and ocular/nasal allergy symptoms in healthy individuals

**Methods:**

This randomized, double-blind, placebo-controlled, parallel-group trial enrolled 120 healthy Japanese adults experiencing cold sensitivity. Subjects consumed edible oils with different ALA contents (1.27 g or 2.03 g/day) or placebo for 12 weeks. Cold sensitivity (blood flow and skin temperature after cold exposure), vascular endothelial function, and allergy symptoms were measured

**Results:**

Skin blood flow and skin surface temperature showed significantly higher values or trends toward higher values in ALA groups during early measurement period following cold exposure (*p* = 0.014 and 0.061, for skin surface temperature of the low- and high-dose groups, respectively). Overall flow-mediated dilation (FMD) analysis showed no significant differences between ALA groups (*p* = 0.128 and *p* = 0.412, for the low- and high-dose groups, respectively); however, subgroup analysis of subjects with pre-intervention values < 6% showed significant improvement (*p* = 0.038 and *p* = 0.043). No group differences were observed in allergy symptoms. Serum ALA concentrations were significantly higher in both ALA groups compared to the control group (*p* < 0.001). High-dose group showed, significantly higher serum EPA and DPA concentrations (*p* = 0.013 and *p* = 0.035, respectively)

**Conclusion:**

ALA intake increased serum n-3 fatty acids and showed beneficial effects on cold sensitivity and endothelial function in Japanese adults without overt diseases.

**Clinical trial registration:**

[https://center6.umin.ac.jp/cgi-open-bin/ctr/ctr_view.cgi?recptno=R000066500], identifier [UMIN000058298].

## 1 Introduction

α-linolenic acid (ALA) is an essential fatty n-3 fatty acid that is found in certain amounts in common vegetable oils. In particular, flaxseed oil and perilla oil are rich in ALA, with ALA accounting for about 50% of the fatty acids in these oils (1). N-3 fatty acids are broadly classified as either marine-derived (primarily eicosapentaenoic acid (EPA) and docosahexaenoic acid (DHA)) or plant-derived (primarily ALA). In regions where access to seafood is limited, ALA serves as an important source of n-3 fatty acids. Compared to other countries, the average intake of n-3 fatty acids among Japanese people is relatively high (2); however, per capita seafood consumption has been declining in recent years, decreasing by approximately 40% over the past 30 years (3). Even within the Japanese population, there are individuals with low n-3 fatty acid intake, and flaxseed oil and perilla oil can serve as useful alternative sources of n-3 fatty acids to seafood. ALA has antioxidant and anti-inflammatory properties (4–6), and observational and intervention studies suggest that ALA may have cardiovascular protective effects, anti-allergic effects, and may improve asthma symptoms (7–9).

Cold sensitivity is a symptom with a high prevalence rate. A large-scale survey of the Japanese population found that the prevalence of cold sensitivity among adults was approximately 3%, with particularly high rates among women and the elderly (10). It has been reported that individuals with cold hypersensitivity exhibit increased stress and impaired lipid metabolism following short-term sleep deprivation compared to those without the condition, and that it also has a negative impact on quality of life (QOL) (11). Improving cold sensitivity is important for enhancing QOL. Previous study has reported that cold stimulation activates brown adipose tissue and increases energy expenditure (12). Because brown adipose tissue plays an important role in maintaining body temperature and energy metabolism, its involvement in alleviating cold sensitivity is conceivable; however, this study focuses on the association between cold sensitivity and improved vascular function. Cold sensitivity is associated with peripheral vascular function, and some reports suggest that impaired vascular endothelial function, as assessed by the reactive hyperemia index, is involved in the cold-sensitivity constitution observed in young women (13). Regarding vascular function, the efficacy of ALA has been reported for brachial-ankle pulse wave velocity (baPWV), an indicator of arterial stiffness, and for blood pressure (14, 15). The efficacy of 0.97 g ALA intake was demonstrated in a 12-week intervention study involving 120 middle-aged and older women, focusing on baPWV (14). Regarding blood pressure, improvements were reported in a 12-week intervention of 44 individuals with high-normal to mild hypertension after consuming 2.6 g of ALA (15). However, regarding the effects of ALA on cold sensitivity, there are only reports involving small numbers of subjects (3 or 7 individuals) (16), and to our knowledge, no peer-reviewed literature exists. Furthermore, the test examples described in that patent involved a relatively short intake period of 13–14 days for the test food containing ALA. Studies monitoring the incorporation of dietary fatty acids into various body tissues have shown that the time for intake to be reflected differs depending on the tissue—approximately 1–2 weeks for cholesterol esters, 1–2 months for red blood cell membranes, and several years for adipose tissue (17). If lipid uptake into cell membranes is related to the mechanism of action, longer-term monitoring may be more effective.

Indicators of vascular function include PWV and flow-mediated dilation (FMD). While PWV measures arterial stiffness in medium and large arteries by assessing the speed at which the pulse wave travels through blood vessels, FMD evaluates the function of vascular endothelial cells, which regulate the movement of vascular smooth muscle (18). FMD, as an indicator of vascular endothelial function, has been suggested to be a useful marker of cardiovascular disease risk (19). In our previous report, we confirmed that in healthy middle-aged and older women, 12 weeks of ALA intake ranging from 0.97 to 2.13 g significantly improved baPWV compared to the control group (14). Although there are reports demonstrating the efficacy of ALA on vascular endothelial function with walnut consumption and in patients with hypercholesterolemia (20, 21), to our knowledge, no studies have confirmed the efficacy of ALA alone in healthy individuals.

Allergic reactions are a type of inflammatory response, and approximately 25% of people in developed countries are affected by them (22). Allergic diseases have been reported to impact daily life, including QOL and presenteeism (23, 24), making them a significant issue in modern society. N-3 fatty acids are known to alter the lipid mediator profile (25), and administration of ALA to mice has been reported to alleviate symptoms of allergic conjunctivitis (26). However, to our knowledge, there are no reports on the efficacy of ALA intake alone for ocular and nasal allergy symptoms in healthy humans.

In this study, we investigated the effects of ALA intake on cold sensitivity, vascular endothelial function, and ocular and nasal allergy symptoms in healthy Japanese adult men and women.

## 2 Materials and methods

### 2.1 Conducting the study, and ethical considerations

This study was conducted by HUMA R&D Co., Ltd. (Tokyo, Japan), a contract research organization, at the Tokyo Shinjuku Clinic (Medical Corporation Taifukukai) under the supervision of physicians. The study was reviewed and approved by the Tokyo Shinjuku Clinic Ethical Review Board (approval number: RD12004TS03, approval date: 16 June 2025). The ethics review process ensured that the study complied with the Declaration of Helsinki, the Ethical Guidelines for Medical and Biological Research Involving Human Subjects (27), and the Act on the Protection of Personal Information (28). Furthermore, to ensure research transparency, the study details were registered with UMIN-CTR prior to study initiation (UMIN-ID: UMIN000058298, URL: https://center6.umin.ac.jp/cgi-open-bin/ctr/ctr_view.cgi?recptno=R000066500). Participants received remuneration to compensate for their participation burden.

### 2.2 Participants

Participants were recruited from a subject registry containing hundreds of thousands of registered individuals and screened based on physical examination, vital signs (systolic blood pressure, diastolic blood pressure, heart rate), height, weight, body mass index (BMI), n-3 fatty acid intake, degree of cold sensitivity, and FMD. Those who met the inclusion criteria, fulfilled none of the exclusion criteria, agreed to adhere to lifestyle and dietary management, and were judged suitable for participation by the study physician were enrolled. Participants were instructed to maintain their usual lifestyle during the study period and to avoid excessive exercise, dieting, overeating, and heavy alcohol consumption. They were also instructed to avoid taking any medications, food supplements, or dietary supplements that could potentially influence antioxidant capacity.

#### 2.2.1 Inclusion criteria

The inclusion criteria were as follows: (a) Males and Females living in Japan aged between 20 and 65 years at the time of written informed consent; (b) individuals with a BMI below 30 kg/m^2^; (c) individuals experiencing cold sensitivity; (d) non-smokers; and (e) individuals with an average alcohol intake of less than 30 g/day.

#### 2.2.2 Exclusion criteria

Exclusion criteria were as follows: (a) individuals with a current or past history of serious diseases (e.g., brain disorders, malignant tumors, immune disorders); (b) individuals undergoing exercise or dietary therapy under medical supervision; (c) individuals with allergies to flaxseed or olive oil, which are ingredients in the test food; (d) individuals with a current or past history of drug or alcohol dependence; (e) individuals with a current or past history of mental or sleep disorders; (f) individuals with significant wounds or inflammation at the measurement site (hand/arm) that would interfere with device measurements; (g) individuals with extremely irregular lifestyles (e.g., night shift work); (h) individuals whose weight has fluctuated by ± 5 kg or more within the past 2 months; (i) individuals regularly taking food supplements, dietary supplements, or medications that affect vascular function or antioxidant capacity (e.g., vitamin E, vitamin C, astaxanthin, beta-carotene, sesamin); and (j) individuals consuming edible oils (medium-chain triglycerides, flaxseed oil, perilla oil, EPA, DHA, olive oil, etc.) three or more days per week for health maintenance purposes.

### 2.3 Sample size

A previous study (14) examining the effects of ALA intake on baPWV demonstrated efficacy at doses of 0.97 g or higher in a trial comprising four groups: one control group and three ALA dose groups, each with 30 subjects. However, a previous study (29) investigating the effect on FMD in hypertensive individuals with metabolic syndrome, involving 41 control participants and 40 treatment participants (1.5 g/day ALA), found no significant difference between groups. Although ALA’s efficacy has been demonstrated for arterial compliance, no positive results were observed for FMD or cold sensitivity measured in this trial. Furthermore, the age ranges of subjects differed between the previous study demonstrating efficacy and this trial. Assuming a moderate difference between the treatment group and the control group and setting the effect size at 0.65, the calculated required sample size was 39 subjects or more. Considering potential dropouts and protocol violations resulting in exclusion from analysis, the sample size for each group was set at 40 subjects. Since this study consists of one control group and two dose-investigation groups, a total of 120 subjects was required.

### 2.4 Study design

This randomized, placebo-controlled, double-blind, parallel-group trial was conducted in healthy Japanese adult males and females. Measurements were performed at a clinic in Tokyo from September to December 2025. A third party not involved in the trial randomly allocated subjects to three groups. The stratification factors were age, sex, BMI, n-3 fatty acid intake, skin blood flow, and FMD. The allocation manager strictly maintained the confidentiality of the allocation list until unblinding to ensure blinding of all other personnel involved in the study.

#### 2.4.1 Specific compliance before test day

Participants were instructed to follow these guidelines from 3 days before to the day before the testing: (a) refrain from strenuous exercise, saunas, hot stone baths, hot springs, and similar activities starting 3 days prior to testing; (b) abstain from alcohol on the day before testing; and (c) maintain their usual sleep duration and quality on the night before testing.

#### 2.4.2 Specific compliance on test day

Participants were instructed to adhere to the following on the day of testing: (a) If the reception time is in the morning, consume a light meal up to 5 h before blood collection and refrain from eating or drinking anything other than water until testing is complete. If the reception time is in the afternoon, consume breakfast and a light meal up to 5 h before blood collection, and refrain from eating or drinking anything other than water until testing is complete; (b) From 3.5 h before blood collection, drink water only in small amounts, totaling no more than 100 mL; (c) On the test day, avoid consuming ginger, garlic, chocolate, or caffeinated beverages; and (d) On the test day, refrain from applying creams, cosmetics, sunscreen, or similar products to the arms or hands until all tests are completed.

### 2.5 Test oils and dietary surveys

The test oils were refined olive oil (Control oil), a blend of flaxseed oil and refined olive oil (Experimental oil (1), and flaxseed oil (Experimental oil (2). From the baseline test day (week 0) until the day before the week 12 test, participants consumed one packet of the test oil (4.08 g/day) in addition to their usual diet. The amounts of each fatty acid contained in one daily serving of each test oil are shown in Table 1.

**TABLE 1:** Daily content of each fatty acid in each test oil.

Fatty acid species	Values	Control oil	Experimental oil 1	Experimental oil 2
Palmitic acid (16:0)	g/day	0.49	0.32	0.21
Palmitoleic acid (16:1)	g/day	0.05	0.02	ND
Stearic acid (18:0)	g/day	0.12	0.15	0.17
Oleic acid (18:1 n-9)	g/day	2.64	1.48	0.76
Vaccenic acid (18:1 n-7)	g/day	0.10	0.05	0.03
Linoleic acid (18:2 n-6)	g/day	0.35	0.49	0.60
ALA (18:3 n-3)	g/day	0.03	1.27	2.03
Arachidic acid (20:0)	g/day	0.02	0.01	< 0.01
Eicosenoic acid (20:1 n-9)	g/day	0.01	0.01	< 0.01
Behenic acid (22:0)	g/day	< 0.01	< 0.01	< 0.01

ALA, α-linolenic acid; ND, not detected.

Dietary intake was assessed at baseline and at week 12 using the brief-type self-administered diet history questionnaire (BDHQ) to calculate nutrient intakes (30).

### 2.6 Lifestyle questionnaire

During the study period, participants recorded their daily lifestyle conditions, including intake of the test food, physical condition, medication use, exercise, alcohol intake, consumption of food supplements and dietary supplements, changes in the intake of foods affecting the study parameters, changes in lifestyle habits, and body weight.

### 2.7 Adverse events

Adverse events were assessed using a lifestyle questionnaire, and body weight was monitored for abnormal fluctuations. When adverse events or abnormal fluctuations occurred, the study physician evaluated the causal relationship with the test food.

### 2.8 Measurements

The primary outcome was cold sensitivity, assessed by skin blood flow and skin surface temperature after cold water exposure. Secondary outcomes included FMD, a questionnaire on ocular and nasal symptoms, and serum lipids. All measurements were performed at baseline (week 0) and at week 12.

#### 2.8.1 Cold sensitivity

Prior to the measurement, participants underwent an 8-min acclimation period under controlled environmental conditions of 24 ± 1°C temperature and 50 ± 5% humidity. Subsequently, each participantan 8erformed at bto the wrist was immersed in 15°C cold water for 1 min, then dried off. Skin blood flow and skin surface temperature were measured immediately after cold water immersion and at 2.5, 5, 7.5, 10, 15, 20, 25, and 30 min thereafter. Measurements were taken at five locations: the center of the dorsal hand (midpoint of the line connecting the base of the index finger and the ulnar styloid process), and near the base of the nails of the index, middle, ring, and little fingers. Skin blood flow was measured using the OMEGAZONE OZ-1 (OMEGAWAVE Inc., Tokyo, Japan), and skin surface temperature was measured using the InfReC R500 (NIPPON AVIONICS Co., Ltd., Kanagawa, Japan). Skin blood flow and skin surface temperature measurements using these devices have been reported in previous studies and are considered valid (31).

#### 2.8.2 FMD

After 15 min of rest in the supine position, a cuff was placed on the right forearm and inflated to systolic blood pressure plus 50 mmHg for 5 min. After the cuff was released, the brachial artery diameter was measured by ultrasound for 2 min, and FMD was calculated using the following formula: [(Maximum dilated diameter–Resting diameter)/Resting diameter] × 100 (%). A general-purpose ultrasound imaging device (UNEXEF18VG) (UNEX Corporation, Aichi, Japan) was used for measurement. The utility and validity of semi-automated FMD measurement devices in this series have been verified in a multicenter prospective study (32).

#### 2.8.3 Eye and nasal symptoms

The Japanese Allergic Conjunctival Diseases QOL Questionnaire (JACQLQ) was used to assess ocular and nasal symptoms. The validity and reliability of this questionnaire have been confirmed in a multicenter study (33).

#### 2.8.4 Fatty acid composition of blood serum and test oils

Serum fatty acid concentrations were measured by gas chromatography after alkaline hydrolysis followed by methyl esterification under acidic conditions (34). Tricosanoic acid was used as an internal standard for quantifying fatty acids in the samples. The EPA:arachidonic acid (AA) ratio (EPA:AA ratio) and n-6:n-3 ratio were calculated. The ω-3 index (O3I) was calculated according to Schuchardt et al. (35). The fatty acid composition of the test oils was analyzed using the same principle as for serum.

### 2.9 Statistical analysis

The actual values and change from baseline (calculated by subtracting baseline values from those at week 12) were analyzed according to the methods specified in the statistical analysis plan prepared prior to data lock.

#### 2.9.1 Energy and nutrient intake

Multiple comparisons between groups were performed for energy intake, nutrient intake, and intake of various n-3 and n-6 fatty acids during the intervention period. Normality was assessed using the Shapiro-Wilk test. When normality was confirmed, homogeneity of variance was assessed using the Bartlett’s test. The Dunnett test was applied when both normality and homogeneity of variance were confirmed; otherwise, the Steel-Dwass test was used.

#### 2.9.2 Cold sensitivity, FMD, eye and nasal symptoms, and blood serum

For cold sensitivity, the average values of skin blood flow and skin surface temperature measured at five locations—the center of the dorsal hand (midpoint of the line connecting the base of the index finger and the ulnar styloid process), and near the base of the nails of the index, middle, ring, and little fingers—were used for analysis. The area under the curve (AUC) from immediately after cold water immersion to each time point was calculated and used for analysis.

For between-group comparisons, values at week 0 were first tested for normality using the Shapiro-Wilk test. If normality was confirmed, homogeneity of variance was assessed using Bartlett’s test. If both normality and homogeneity of variance were confirmed, multiple comparisons were performed using Dunnett’s method; otherwise, Steel’s method was used. After confirming no significant between-group differences in week 0 values, between-group comparisons for week 12 values were conducted as follows: first, normality and homogeneity of variance were assessed using the Shapiro-Wilk and Bartlett’s tests. If both assumptions were met, multiple comparisons were performed using Williams’ method; otherwise, the Shirley-Williams’ method was used. For variables showing significant between-group differences at week 0, normality and homogeneity of variance for week 12 values were first assessed with the Shapiro-Wilk test and Bartlett’s test. If these assumptions were not met, data were standardized. Subsequently, Dunnett’s test was used for between-group comparisons of week 12 values, with week 0 values as covariates.

For within-group comparisons, normality was tested using the Shapiro-Wilk test. If normality was confirmed, paired *t*-tests were performed; otherwise, Wilcoxon signed-rank tests were used.

For vascular endothelial function, a stratified subgroup analysis was performed by categorizing participants based on their pre-intervention vascular endothelial function, and the aforementioned analyses were conducted. The threshold values for FMD vary among guidelines and studies, with suggested cutoff points such as 7.1, 6.5, and 6% (36–38). Due to considerable variability in FMD measurement methods, reproducibility and comparisons between studies are challenging (39), and there is currently no internationally established consensus. However, cutoff values in the range of approximately 4–7% are generally accepted. A study involving healthy Japanese adults has reported that the incidence of coronary artery disease is virtually nonexistent among individuals with an FMD of 6% or higher (38). Therefore, in this study, FMD values of 6% or higher were considered normal, and a stratified analysis using 6% as the cutoff was conducted.

Basic statistical measures were calculated using Microsoft Excel for Office365 MSO (Microsoft Japan Co., Ltd., Tokyo, Japan), and statistical analyses were performed using R statistical software, v4.1.0 for Windows (R Core Team, Vienna, Austria). In all significance tests, a *p*-value of < 0.05 was considered statistically significant, and 0.05 < *p*-value < 0.1 was considered indicative of a statistical trend.

## 3 Results

### 3.1 Participants

**FIGURE 1:**
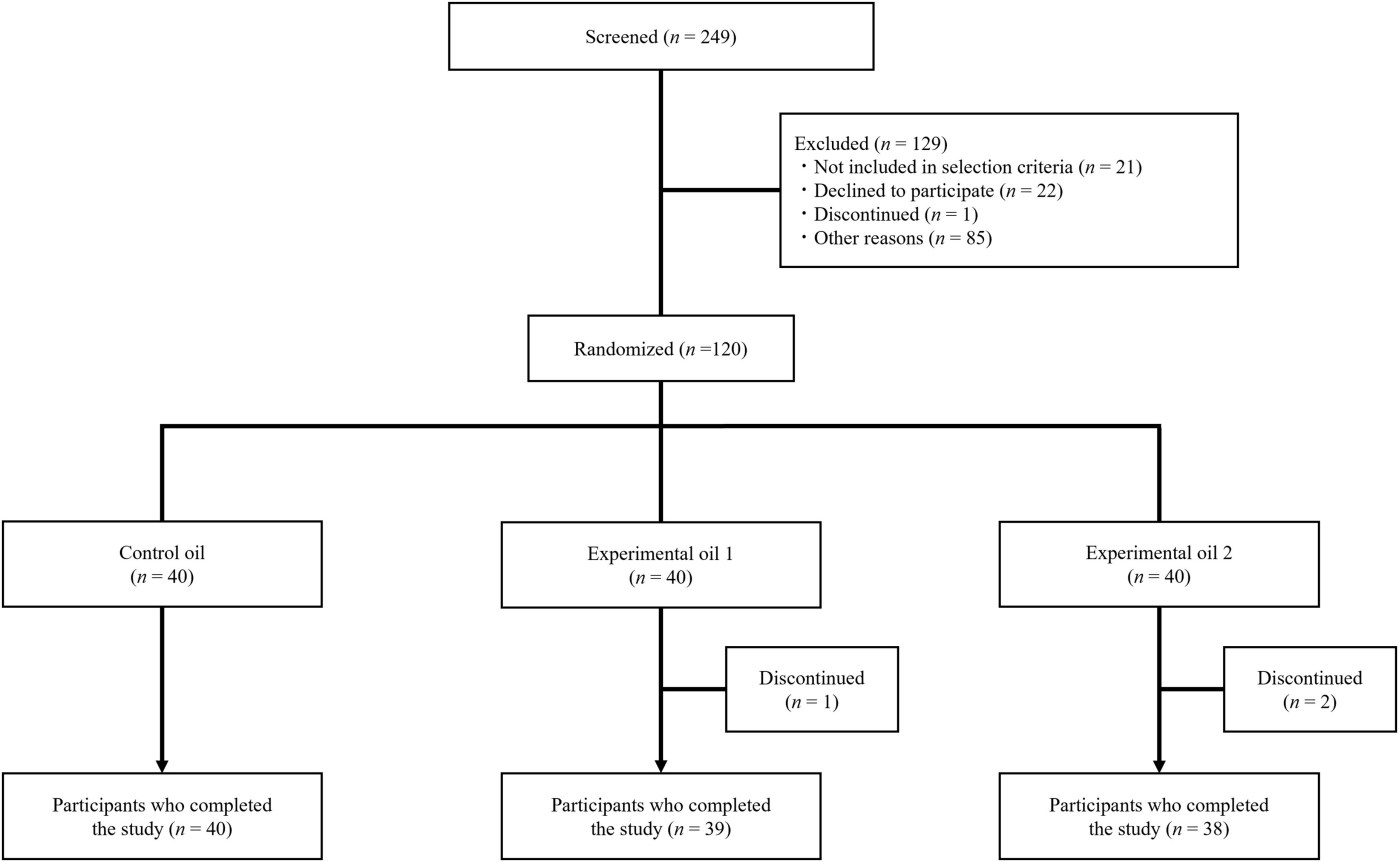
Flowchart of the study.

Among the 249 individuals assessed for eligibility, 120 participants were enrolled in the trial. These participants were randomly assigned to three groups (control oil group, experimental oil 1 group, experimental oil 2 group), with 40 participants in each group. During the intervention period, one participant in the experimental oil 1 group and two participants in the experimental oil 2 group discontinued the study. Excluding those removed according to the statistical analysis plan, 117 participants were included in the Per Protocol Set (PPS) for analysis (Figure 1). Baseline characteristics of the analyzed participants are shown in Table 2.

**TABLE 2:** Baseline clinical characteristics of the participants^1^.

Characteristics	Values	Control oil	Experimental oil 1	Experimental oil 2
Age	Years	46.0 ± 12.2	45.0 ± 12.0	46.7 ± 13.6
Height	cm	162.1 ± 7.5	164.3 ± 9.4	162.0 ± 6.8
Body weight	kg	54.4 ± 10.9	56.2 ± 10.2	53.5 ± 9.6
BMI	kg/m^2^	20.5 ± 3.0	20.7 ± 2.8	20.2 ± 2.6
Sex	Male	10	12	12
	Female	30	27	26

^1^Values are shown as mean ± standard deviation. BMI, body mass index.

### 3.2 Adverse events

No adverse events attributable to the test oils were observed.

### 3.3 Cold sensitivity

The results of skin blood flow and skin surface temperature measurements during the study period are shown in Table 3. Regarding changes from baseline in skin blood flow, the experimental oil 1 group showed significantly higher AUC values compared to the control oil group for 0–2.5 min and 0–5 min after cold water immersion (*p* = 0.014 and *p* = 0.028, respectively), and a trend toward higher AUC values for 0–7.5 min (*p* = 0.073). No significant differences between groups were observed after 10 min of cold water immersion. Regarding changes from baseline in skin surface temperature, the experimental oil 1 group showed significantly higher AUC values compared to the control oil group for 0–2.5 min and 0–5 min (*p* = 0.020 and *p* = 0.039, respectively), and a trend toward higher AUC values for 0–7.5 min (*p* = 0.088). In the experimental oil 2 group, the AUC values for 0–2.5 min and 0–5 min after cold water immersion showed a trend toward higher values compared to the control oil group (*p* = 0.061 and *p* = 0.090, respectively). No significant differences between groups were observed after 10 min of cold water immersion.

**TABLE 3:** Blood flow and temperature of hand after cold water immersion during the intervention period^1^.

Outcomes	Values	Time interval	Week	Control oil		Experimental oil 1		Experimental oil 2	
				Actual value	Change value	Actual value	Change value	Actual value	Change value
Blood flow		0–2.5 min	0	29.71 ± 3.44	7.13 ± 3.24^†^	28.82 ± 3.96	9.15 ± 3.48**^†^	29.42 ± 2.70	8.43 ± 3.94^†^
			12	36.84 ± 3.95^†^		37.97 ± 3.83^†^		37.85 ± 3.86^†^	
		0–5 min	0	59.71 ± 7.38	15.07 ± 7.67^†^	57.82 ± 8.30	19.02 ± 7.53**^†^	59.25 ± 5.66	17.47 ± 8.29^†^
			12	74.78 ± 8.75^†^		76.84 ± 8.80^†^		76.72 ± 8.20^†^	
		0–7.5 min	0	90.23 ± 11.62	24.60 ± 12.76^†^	29.77 ± 12.33*^†^	87.71 ± 12.63	90.25 ± 8.86	27.28 ± 12.77^†^
			12	114.83 ± 14.74^†^		117.48 ± 14.80^†^		117.53 ± 12.63^†^	
		0–10 min	0	121.34 ± 16.16	35.09 ± 18.79^†^	118.48 ± 16.70	41.07 ± 17.35^†^	122.11 ± 12.13	38.01 ± 17.41^†^
			12	156.44 ± 22.08^†^		159.55 ± 21.17^†^		160.12 ± 17.38^†^	
		0–15 min	0	184.67 ± 25.23	57.00 ± 32.01^†^	180.79 ± 24.73	65.45 ± 28.17^†^	186.66 ± 18.76	61.88 ± 28.37^†^
			12	241.67 ± 38.06^†^		246.24 ± 35.79^†^		248.54 ± 28.63^†^	
		0–20 min	0	248.53 ± 34.35	80.22 ± 45.16^†^	243.62 ± 32.42	91.42 ± 41.92^†^	251.66 ± 25.73	88.21 ± 41.92^†^
			12	328.75 ± 54.17^†^		335.04 ± 52.73^†^		339.87 ± 41.19^†^	
		0–25 min	0	312.40 ± 43.32	104.38 ± 57.57^†^	306.47 ± 39.72	118.14 ± 56.44^†^	316.78 ± 32.86	115.54 ± 54.52^†^
			12	416.78 ± 69.81^†^		424.61 ± 69.90^†^		432.32 ± 52.58^†^	
		0–30 min	0	376.25 ± 51.87	128.08 ± 69.17^†^	368.89 ± 47.24	144.65 ± 69.84^†^	381.59 ± 40.06	142.36 ± 65.23^†^
			12	504.34 ± 84.07^†^		513.54 ± 85.73^†^		523.94 ± 63.42^†^	
Temperature	°C ⋅ min	0–2.5 min	0	55.87 ± 6.04	−3.07 ± 5.10^†^	53.76 ± 4.48	−1.14 ± 4.68**	55.50 ± 6.12	−1.91 ± 4.81*^†^
			12	52.81 ± 4.14^†^		52.62 ± 3.30		53.59 ± 4.12^†^	
		0–5 min	0	116.96 ± 14.91	−6.55 ± 13.36^†^	112.53 ± 12.31	−2.41 ± 12.43**	116.54 ± 15.14	−3.74 ± 11.63*
			12	110.41 ± 10.89^†^		110.12 ± 9.62		112.80 ± 11.57	
		0–7.5 min	0	180.99 ± 24.59	−8.17 ± 21.73^†^	174.52 ± 21.31	−3.08 ± 20.66*	181.33 ± 24.50	−4.81 ± 19.02
			12	172.82 ± 19.71^†^		171.44 ± 18.37		176.52 ± 20.30	
		0–10 min	0	247.15 ± 34.58	−8.51 ± 29.77^†^	238.79 ± 30.46	−3.28 ± 28.37	249.41 ± 33.54	−5.68 ± 26.29
			12	238.64 ± 29.64^†^		235.51 ± 27.90		243.73 ± 29.08	
		0–15 min	0	383.85 ± 54.19	−9.32 ± 45.77^†^	371.25 ± 48.68	−2.65 ± 42.42	391.06 ± 50.48	−7.40 ± 40.89
			12	374.53 ± 49.78^†^		368.60 ± 46.99		383.66 ± 46.24	
		0–20 min	0	523.92 ± 73.15	−10.32 ± 61.73	506.56 ± 66.66	−1.02 ± 55.74	536.34 ± 66.82	−9.07 ± 56.13
			12	513.60 ± 69.40		505.54 ± 65.91		527.27 ± 63.13	
		0–25 min	0	665.53 ± 91.61	−10.92 ± 77.89	643.47 ± 84.25	1.01 ± 69.44	683.03 ± 83.11	−10.38 ± 71.67
			12	654.61 ± 88.28		644.48 ± 84.57		672.65 ± 79.89	
		0–30 min	0	808.16 ± 109.39	−11.04 ± 94.15	781.43 ± 101.11	3.49 ± 83.48	830.42 ± 99.06	−11.64 ± 86.26
			12	797.12 ± 106.30		784.92 ± 102.93		818.78 ± 96.46	

^1^Values are shown as mean ± standard deviation.

^**^Significantly different from the control group (*p* < 0.05, Williams’ multiple comparison test or Shirley-Williams’ multiple comparison test). *Marginally different from the control group (*p* < 0.1, Williams’ multiple comparison test or Shirley-Williams’ multiple comparison test).

† Significantly different from the baseline (*p* < 0.05, paired *t*-test or Wilcoxon signed-rank test).

### 3.4 Nutrient intake

The daily nutrient intake (excluding the test oils) during the intervention is shown in Table 4. No significant differences were observed between groups in the intake of any nutrient.

**TABLE 4:** Daily nutrient intake during the intervention period (excluding test oils)^1^.

Nutrients	Values	Week	Control oil	Experimental oil 1	Experimental oil 2
Energy	kcal	0	1411.5 ± 396.0	1497.5 ± 432.2	1463.7 ± 459.7
		12	1344.1 ± 479.0	1393.9 ± 343.1	1361.1 ± 459.2
Carbohydrate	g	0	182.3 ± 56.5	190.7 ± 54.8	190.5 ± 52.6
		12	177.1 ± 67.8	178.9 ± 44.1	175.8 ± 55.4
Protein	g	0	54.4 ± 17.7	56.8 ± 17.4	55.6 ± 21.5
		12	53.3 ± 21.1	54.6 ± 16.9	53.6 ± 22.0
Fat	g	0	47.8 ± 15.6	51.7 ± 20.4	49.4 ± 20.2
		12	43.5 ± 16.5	47.4 ± 16.4	45.5 ± 18.9
ALA (18:3 n-3)	mg	0	1515.4 ± 564.4	1623.7 ± 679.9	1496.7 ± 544.7
		12	1388.7 ± 549.4	1498.2 ± 551.7	1454.7 ± 635.6
EPA (20:5 n-3)	mg	0	177.3 ± 120.2	213.5 ± 137.3	204.1 ± 142.4
		12	185.7 ± 152.8	205.9 ± 152.7	200.5 ± 185.7
DPA (22:5 n-3)	mg	0	56.4 ± 32.2	65.1 ± 36.1	64.4 ± 41.1
		12	59.2 ± 40.8	64.3 ± 40.0	62.7 ± 48.4
DHA (22:6 n-3)	mg	0	330.5 ± 193.6	387.8 ± 208.9	372.7 ± 239.8
		12	339.6 ± 247.7	370.8 ± 235.0	358.9 ± 295.4
Linoleic acid (18:2 n-6)	mg	0	9747.4 ± 3407.4	10364.2 ± 3836.8	9622.6 ± 3623.6
		12	8933.4 ± 3407.6	9546.3 ± 3135.4	9182.7 ± 3863.8
γ-linolenic acid (18:3 n-6)	mg	0	6.8 ± 4.0	7.4 ± 5.4	7.2 ± 4.9
		12	4.6 ± 3.3	4.7 ± 3.8	4.9 ± 5.4
Dihomo-γ-linolenic acid (20:3 n-6)	mg	0	25.7 ± 9.0	27.1 ± 11.3	26.8 ± 13.4
		12	23.8 ± 10.5	25.2 ± 9.9	24.7 ± 10.6
Arachidonic acid (20:4 n-6)	mg	0	142.0 ± 52.7	153.2 ± 59.9	149.4 ± 84.1
		12	134.5 ± 61.5	144.5 ± 57.7	136.9 ± 61.5
n-3 fatty acids	mg	0	2.1 ± 0.8	2.4 ± 0.9	2.2 ± 0.9
		12	2.0 ± 0.9	2.2 ± 0.9	2.1 ± 1.1
n-6 fatty acids	mg	0	10.0 ± 3.5	10.6 ± 3.9	9.9 ± 3.7
		12	9.2 ± 3.5	9.8 ± 3.2	9.4 ± 4.0

^1^Values are shown as mean ± standard deviation. ALA, α-linolenic acid.

### 3.5 FMD

The results of FMD measurements during the study period are shown in Table 5. In the analysis of all participants, no significant differences were observed between groups (for experimental oil 1 and experimental oil 2 groups: actual values: *p* = 0.588 and *p* = 0.974, respectively; change values: *p* = 0.128 and *p* = 0.412, respectively). In a subgroup analysis of subjects with pre-intervention FMD values below 6% (*n* = 18, 22, and 17 in the control, experimental oil 1, and experimental oil 2 groups, respectively), both experimental oil 1 and 2 groups showed significant improvement compared to the control group (*p* = 0.038 and *p* = 0.043, respectively).

**TABLE 5:** FMD values during the intervention period^1^.

	Values	Week	Control oil		Experimental oil 1		Experimental oil 2	
			Actual value	Change value	Actual value	Change value	Actual value	Change value
All participants (*n* = 117)	%	0	6.15 ± 2.65	0.69 ± 2.90	5.53 ± 2.78	1.72 ± 2.94^†^	6.07 ± 3.35	0.61 ± 3.49
		12	6.84 ± 3.11		7.25 ± 2.73^†^		6.68 ± 3.13	
Participants with pre-intervention FMD below 6.0% (*n* = 57)	%	0	5.20 ± 2.23	−0.49 ± 2.63	5.26 ± 2.27	1.31 ± 2.13**^†^	4.19 ± 2.43	1.46 ± 3.14**
		12	4.71 ± 2.46		6.56 ± 2.68*^†^		5.65 ± 3.29	

^1^Values are shown as mean ± standard deviation.

**Significantly different from the control group (*p* < 0.05, Williams’ multiple comparison test or Shirley-Williams’ multiple comparison test). *Marginally different from the control group (*p* < 0.1, Williams’ multiple comparison test or Shirley-Williams’ multiple comparison test).

† Significantly different from the baseline (*p* < 0.05, paired *t*-test or Wilcoxon signed-rank test). FMD, flow mediated dilation.

### 3.6 Ocular and nasal symptoms

The results for ocular and nasal symptoms during the intervention period are shown in Table 6. No significant differences were observed between the experimental groups and the control group.

**TABLE 6:** Results of JACQLQ during the intervention period^1^.

Outcomes	Week	Control oil		Experimental oil 1		Experimental oil 2	
		Actual value	Change value	Actual value	Change value	Actual value	Change value
Itchy eyes	0	0.13 ± 0.33	0.15 ± 0.43	0.21 ± 0.41	0.24 ± 0.43	0.24 ± 0.43	0.00 ± 0.52
	12	0.13 ± 0.33		0.00 ± 0.32		−0.05 ± 0.56	
Foreign body sensation	0	0.20 ± 0.46	−0.03 ± 0.48	0.23 ± 0.54	−0.08 ± 0.58	0.18 ± 0.46	0.00 ± 0.52
	12	0.18 ± 0.45		0.15 ± 0.67		0.18 ± 0.39	
Hyperemia	0	0.05 ± 0.22	0.03 ± 0.36	0.13 ± 0.41	0.10 ± 0.50	0.11 ± 0.31	0.05 ± 0.23
	12	0.08 ± 0.27		0.23 ± 0.58		0.16 ± 0.37	
Watery eyes	0	0.10 ± 0.30	0.03 ± 0.42	0.15 ± 0.37	0.21 ± 0.70	0.05 ± 0.23	0.16 ± 0.68
	12	0.13 ± 0.33		0.15 ± 0.37		0.00 ± 0.46	
Eye discharge	0	0.38 ± 0.70	−0.15 ± 0.58	0.26 ± 0.44	0.08 ± 0.66	0.18 ± 0.39	0.03 ± 0.54
	12	0.23 ± 0.42		0.33 ± 0.58		0.21 ± 0.41	
Runny nose	0	0.10 ± 0.38	0.28 ± 0.64^†^	0.13 ± 0.41	0.08 ± 0.58	0.05 ± 0.23	0.21 ± 0.47^†^
	12	0.38 ± 0.74^†^		0.21 ± 0.47		0.26 ± 0.50^†^	
Sneezing	0	0.13 ± 0.33	0.13 ± 0.52	0.21 ± 0.47	0.13 ± 0.52	0.26 ± 0.45	0.00 ± 0.52
	12	0.25 ± 0.49		0.33 ± 0.53		0.26 ± 0.45	
Nasal congestion	0	0.15 ± 0.36	0.03 ± 0.58	0.08 ± 0.27	0.03 ± 0.36	0.16 ± 0.37	−0.08 ± 0.43
	12	0.18 ± 0.55		0.10 ± 0.31		0.08 ± 0.27	
Itchy nose	0	0.10 ± 0.30	−0.03 ± 0.42	0.10 ± 0.31	−0.05 ± 0.22	0.16 ± 0.37	−0.05 ± 0.23
	12	0.08 ± 0.35		0.05 ± 0.22		0.11 ± 0.31	

^1^Values are shown as mean ± standard deviation. BMI, body mass index.

† Significantly different from the baseline (*p* < 0.05, paired *t*-test or Wilcoxon signed-rank test). JACQLQ, Japanese allergic conjunctival diseases; QOL, Questionnaire.

### 3.7 Body weight, BMI, and serum lipids

Body composition and serum lipid values during the intervention period are shown in Table 7. No significant differences were observed between groups in body weight, BMI, total cholesterol, LDL cholesterol, HDL cholesterol, or triglycerides.

**TABLE 7:** Body weight, BMI, blood pressure and serum lipids during the intervention period^1^.

Outcomes	Values	Week	Control oil	Experimental oil 1	Experimental oil 2
Body weight	kg	0	55.0 ± 11.1	56.8 ± 10.6	54.0 ± 9.6
		12	55.5 ± 11.0^†^	57.1 ± 10.4^†^	54.1 ± 9.8
BMI	kg/m^2^	0	20.8 ± 3.0	20.9 ± 2.9	20.4 ± 2.6
		12	21.0 ± 3.1^†^	21.0 ± 2.8^†^	20.5 ± 2.7
Systolic blood pressure	mmHg	0	104.8 ± 15.0	107.1 ± 11.2	108.9 ± 12.3*
		12	105.8 ± 13.9	107.6 ± 10.8	108.1 ± 11.7
Diastolic blood pressure	mmHg	0	72.0 ± 9.1	73.8 ± 8.4	74.6 ± 8.6
		12	72.2 ± 9.0	74.0 ± 9.0	74.4 ± 9.0
TC	mg/dL	0	193.5 ± 30.5	186.2 ± 25.7	196.9 ± 34.2
		12	195.3 ± 30.4	185.9 ± 26.5	200.2 ± 36.5
LDL-C	mg/dL	0	109.3 ± 28.4	102.8 ± 22.7	110.0 ± 29.2
		12	112.1 ± 26.7	103.3 ± 22.1	111.9 ± 29.9
HDL-C	mg/dL	0	67.7 ± 13.3	71.6 ± 14.9	73.2 ± 17.2
		12	69.8 ± 12.6^†^	71.5 ± 15.0	75.3 ± 16.5
TG	mg/dL	0	99.8 ± 121.8	71.1 ± 31.1	83.0 ± 47.9
		12	80.5 ± 47.4^†^	66.7 ± 28.9	78.9 ± 47.1

^1^Values are shown as mean ± standard deviation.

**Significantly different from the control group (*p* < 0.05, Williams’ multiple comparison test or Shirley-Williams’ multiple comparison test). *Marginally different from the control group (*p* < 0.1, Williams’ multiple comparison test or Shirley-Williams’ multiple comparison test).

† Significantly different from the baseline (*p* < 0.05, paired *t*-test or Wilcoxon signed-rank test). BMI, body mass index; HDL, high-density lipoprotein; LDL, low density lipoprotein; TC, total cholesterol; TG, triglyceride.

### 3.8 Fatty acid composition of blood serum

Serum n-3 fatty acid concentrations during the study period are shown in Table 8. Serum ALA concentrations at week 12 were significantly higher in both experimental groups compared to the control group (actual values: *p* = 0.005 and *p* < 0.001, respectively; change values: *p* < 0.001 for both groups). In the experimental oil 2 group, the actual values of serum EPA and DPA concentrations and the EPA:AA ratio at week 12 were significantly higher than those in the control group (*p* = 0.013, *p* = 0.035, and *p* = 0.014, respectively), and serum DHA concentration showed a trend toward higher values (*p* = 0.071). The actual values of the n-6:n-3 ratio at week 12 were significantly lower in both experimental groups compared to the control group (*p* = 0.037 and *p* = 0.003, respectively). No significant between-group differences were observed in the O3I.

**TABLE 8:** Fatty acid composition in the blood serum during the intervention period^1,2^.

Fatty acid species	Values	Week	Control oil		Experimental oil 1		Experimental oil 2	
			Actual value	Change value	Actual value	Change value	Actual value	Change value
Lauric acid (12:0)	μg/mL	0	3.11 ± 3.80	−0.89 ± 2.78	2.55 ± 4.08	−0.91 ± 4.52	1.67 ± 1.67	0.30 ± 2.32
		12	2.22 ± 2.17		1.64 ± 2.01**		1.98 ± 2.00	
Myristic acid (14:0)	μg/mL	0	21.67 ± 17.17	−4.28 ± 12.19	18.27 ± 10.72	−3.33 ± 8.50^†^	18.05 ± 7.56	0.39 ± 8.36
		12	17.38 ± 9.53		14.93 ± 7.19^†^		18.44 ± 11.07	
Palmitic acid (16:0)	μg/mL	0	652.74 ± 301.48	−30.16 ± 217.26	586.34 ± 110.36	1.86 ± 83.27	661.96 ± 136.71	2.51 ± 94.71
		12	622.59 ± 159.70		588.20 ± 119.25		664.46 ± 166.37*	
Palmitoleic acid (16:1 n-7)	μg/mL	0	46.07 ± 27.32	−7.38 ± 19.97^†^	42.48 ± 26.08	−6.29 ± 13.98^†^	48.08 ± 19.84	−2.66 ± 13.28
		12	38.69 ± 16.27^†^		36.19 ± 16.17^†^		45.43 ± 24.67	
Stearic acid (18:0)	μg/mL	0	205.02 ± 59.71	−5.83 ± 42.22	194.56 ± 29.83	2.50 ± 27.56	204.34 ± 31.26	2.92 ± 22.81
		12	199.18 ± 35.65		197.06 ± 37.34		207.26 ± 37.06	
Oleic acid (18:1 n-9)	μg/mL	0	585.55 ± 447.96	−49.13 ± 338.75	481.85 ± 120.95	−16.89 ± 74.96	557.12 ± 184.86	−11.89 ± 113.77
		12	536.42 ± 182.98		464.95 ± 122.41*		545.22 ± 208.72	
Linoleic acid (18:2 n-6)	μg/mL	0	888.97 ± 255.69	−22.02 ± 225.60	826.15 ± 131.89	6.80 ± 157.43	892.87 ± 169.81	−14.95 ± 140.53
		12	866.95 ± 166.15		832.95 ± 130.71		877.91 ± 181.16	
γ-linolenic acid (18:3 n-6)	μg/mL	0	9.30 ± 5.16	−0.77 ± 3.73	8.01 ± 4.30	−0.71 ± 3.11	8.16 ± 5.44	−0.58 ± 3.56
		12	8.54 ± 4.26		7.30 ± 3.53		7.59 ± 4.60	
ALA (18:3 n-3)	μg/mL	0	20.26 ± 20.55	−4.17 ± 19.99	16.57 ± 6.94	5.84 ± 10.67**^†^	17.5 ± 8.00	9.71 ± 9.76**^†^
		12	16.09 ± 5.99		22.41 ± 12.71**^†^		27.21 ± 14.32**^†^	
Arachidic acid (20:0)	μg/mL	0	7.31 ± 2.30	−0.18 ± 1.93	6.93 ± 1.02	0.05 ± 0.65	7.31 ± 1.13	0.18 ± 0.69
		12	7.13 ± 1.15		6.98 ± 0.99		7.49 ± 1.29	
Eicosenoic acid (20:1 n-9)	μg/mL	0	4.70 ± 3.91	−0.52 ± 3.28	4.13 ± 1.21	0.01 ± 1.46	4.59 ± 1.59	−0.09 ± 1.65
		12	4.19 ± 1.18		4.13 ± 1.60		4.50 ± 1.47	
Eicosadienoic acid (20:2 n-6)	μg/mL	0	8.50 ± 3.01	−0.14 ± 2.54	8.40 ± 1.71	−0.44 ± 1.60	8.96 ± 1.81	−0.24 ± 1.53
		12	8.36 ± 2.02		7.97 ± 2.24		8.72 ± 2.29	
Dihomo-γ-linolenic acid (20:3 n-6)	μg/mL	0	31.07 ± 10.54	0.56 ± 8.00	30.18 ± 7.54	−0.36 ± 8.23	31.15 ± 10.80	−0.14 ± 6.77
		12	31.63 ± 9.43		29.82 ± 9.32		31.01 ± 10.96	
Arachidonic acid (20:4 n-6)	μg/mL	0	177.53 ± 48.37	−2.07 ± 24.52	175.31 ± 35.65	−5.71 ± 21.01^†^	180.76 ± 44.63	−1.49 ± 27.87
		12	175.46 ± 44.39		169.60 ± 38.15		179.26 ± 43.37	
EPA (20:5 n-3)	μg/mL	0	31.71 ± 25.44	−5.55 ± 21.60	35.11 ± 26.62	2.05 ± 18.15	37.22 ± 31.11	0.41 ± 32.64
		12	26.16 ± 14.54		37.16 ± 27.05		37.62 ± 22.52**	
Behenic acid (22:0)	μg/mL	0	19.35 ± 3.75	0.48 ± 2.59	17.89 ± 2.96	0.74 ± 2.04^†^	19.51 ± 3.53	0.39 ± 2.12
		12	19.83 ± 3.58		18.62 ± 3.03^†^		19.89 ± 3.98	
DPA (22:5 n-3)	μg/mL	0	14.46 ± 5.09	−1.23 ± 3.43	14.42 ± 4.81	−0.06 ± 3.31	15.13 ± 3.82	0.69 ± 3.39**
		12	13.23 ± 3.68		14.36 ± 4.12		15.82 ± 4.05**	
Lignoceric acid (24:0)	μg/mL	0	16.64 ± 3.50	0.97 ± 2.18^†^	15.65 ± 2.70	1.57 ± 2.51^†^	17.08 ± 3.18	1.54 ± 2.16^†^
		12	17.60 ± 3.38^†^		17.22 ± 2.55^†^		18.63 ± 3.85^†^	
DHA (22:6 n-3)	μg/mL	0	82.24 ± 26.24	−2.08 ± 19.34	84.54 ± 28.19	−0.29 ± 13.62	95.55 ± 33.14	−4.32 ± 25.28
		12	80.16 ± 20.95		84.25 ± 27.96		91.23 ± 27.57*	
Nervonic acid (24:1 n-9)	μg/mL	0	30.47 ± 5.25	0.21 ± 4.11	31.95 ± 4.41	0.36 ± 3.79	34.54 ± 5.62**	1.51 ± 4.88**^†^
		12	30.68 ± 4.92		32.31 ± 4.82		36.05 ± 5.46**	
EPA:AA ratio		0	0.18 ± 0.15	−0.03 ± 0.12	0.20 ± 0.13	0.02 ± 0.12	0.20 ± 0.15	0.01 ± 0.18
		12	0.16 ± 0.10		0.22 ± 0.15*		0.21 ± 0.13**	
n-6:n-3 ratio		0	8.87 ± 2.92	0.21 ± 2.04	8.28 ± 2.87	−0.41 ± 2.18	8.34 ± 3.50	−1.00 ± 3.03
		12	9.08 ± 2.49		7.88 ± 2.64**		7.34 ± 2.41**^†^	
O3I		0	6.83 ± 1.97	−0.22 ± 1.53	7.24 ± 1.81	0.14 ± 1.49	7.44 ± 2.53	−0.03 ± 2.46
		12	6.61 ± 1.48		7.37 ± 2.04		7.41 ± 2.25	

^1^Values are shown as mean ± standard deviation.

^2^Weight% of total fatty acids in the fraction.

^**^Significantly different from the control group (*p* < 0.05, Williams’ multiple comparison test or Shirley-Williams’ multiple com-parison test). *Marginally different from the control group (*p* < 0.1, Williams’ multiple comparison test or Shirley-Williams’ multiple comparison test).

† Significantly different from the baseline (*p* < 0.05, paired *t*-test or Wilcoxon signed-rank test). ALA, α-linolenic acid; DHA, docosahexaenoic acid; DPA, docosapentaenoic acid; EPA, eicosapentaenoic acid; O3I, ω-3 index.

## 4 Discussion

In this study, we investigated the effects of continuous 12-week intake of oils containing ALA on cold sensitivity, vascular endothelial function, and ocular and nasal symptoms in healthy Japanese adults.

Regarding cold sensitivity, ALA intake groups showed significant improvements in skin blood flow and skin surface temperature compared to the control group (Table 3). The mechanisms underlying these effects are thought to include improved vascular function, regulation of vasoconstriction, and enhanced blood fluidity. When the extremities are exposed to local cold, the release of neurotransmitters from the sympathetic nervous system to the muscular layer of arteriovenous anastomoses decreases rapidly, leading to cold-induced vasodilation (40). The autonomic nervous system and vascular function are involved in adaptation to local cold. One report suggests that reduced vascular endothelial function is involved in the cold-sensitivity constitution observed in young women (13). ALA improves baPWV, an index of arterial stiffness (14). ALA may have improved cold sensitivity through improved vascular function. Additionally, ALA selectively inhibits prostanoid TP receptors involved in vascular constriction (41), suggesting that ALA may have contributed to cold sensitivity improvement by regulating vasoconstriction as a bioactive substance. There are also reports that n-3 fatty acids improve red blood cell deformability and reduce blood viscosity (42). Red blood cell deformability influences blood flow (43). Previous studies have reported that fish oil alleviates Raynaud’s phenomenon, and in this study, it is possible that EPA and DHA—metabolites from ALA—also contributed to the improvement in cold sensitivity (44). The cold sensitivity improvement observed in this study may be related to enhanced red blood cell deformability and reduced blood viscosity by ALA and its metabolites. ALA intake groups showed significantly higher values than the control group for up to 5 min after cold water exposure, with a tendency toward higher values persisting until 7.5 min. No significant between-group differences were observed thereafter. Since significant improvements in skin blood flow and skin surface temperature were observed early after ALA intake, ALA may have facilitated early recovery of blood flow and skin surface temperature after exposure. The lack of differences after 10 min may be due to the conditions used in this study, such as 1-min immersion in 15°C cold water; previous research (45) also reported no significant differences after early recovery. This may be because skin blood flow and skin surface temperature recovered over time in the control group as well, diminishing between-group differences, and because large measurement variability made statistical significance difficult to detect.

Regarding vascular endothelial function, the overall analysis revealed no significant differences between the experimental groups and the control group (Table 5). A large-scale cross-sectional study of the general Japanese population reported that FMD shows a negative correlation with age and BMI (46). In this study, 31.6% of participants were young adults (aged 39 or younger), and the mean BMI was 20.5, within the normal weight range. In our previous study evaluating ALA efficacy on baPWV, stratified analysis revealed that ALA was effective in the older age group (55–64 years) but not in the younger age group (45–54 years) (14). Because this study included many younger individuals with low cardiovascular disease risk and low risk of reduced vascular endothelial function, the overall analysis may not have adequately assessed ALA efficacy in subjects with reduced vascular function. However, in subjects with low pre-intervention FMD (<6%), the experimental groups showed significantly higher FMD values at week 12 compared to the control group (Table 5). FMD reference values vary across guidelines and studies (36–38). Significant variability in FMD measurement methods makes reproducibility and between-study comparisons difficult (39). Although approximately 4–7% is generally used as the cutoff value, no internationally agreed-upon cutoff exists, and no established criteria exist for definitive diagnosis (37). In this study, we set the cutoff value at 6% based on a study of healthy Japanese individuals (38). The results suggest that ALA intake is effective for individuals with impaired vascular endothelial function. ALA efficacy on FMD may involve combined antioxidant, anti-inflammatory, and blood pressure-lowering effects. Endothelial dysfunction causes oxidative stress and inflammation of the blood vessel wall, leading to further deterioration of vascular function (47). Anti-inflammatory agents protect endothelial cells (48). The antioxidant and anti-inflammatory effects of ALA may have contributed to FMD improvement. ALA exhibits blood pressure-lowering effects by inhibiting angiotensin-converting enzyme (ACE) activity and increasing the vasodilators prostacyclin (PGI2) and nitric oxide (15, 49, 50). Since FMD correlates negatively with blood pressure (46), ALA may have improved FMD through its ACE-inhibitory function. However, in this study, no significant difference in blood pressure was observed between the experimental groups and the control group. This is because the subjects’ mean pre-intervention blood pressure was within the optimal blood pressure range (51), making further ALA-induced reduction unlikely. Multiple reports describe the effects of dietary and exercise interventions on FMD. A meta-analysis of exercise interventions in obese adults found that, across 12 studies incorporating resistance training and aerobic exercise, the average FMD change was 1.17% in the intervention group and 0.56% after subtracting the control group value (52). A systematic review of the Mediterranean dietary pattern found 1.66% FMD improvement (53). In this study’s stratified analysis, ALA improved FMD by 1.31–1.46% in the intervention group and by 1.80–1.95% after subtracting the control group value, representing a substantial effect for a single food component intervention. Efficacy was demonstrated at relatively low doses, and implementation appears straightforward regarding adoption ease and side effect risk. The stratified analysis results in individuals with decreased vascular endothelial function suggest potent ALA effects on FMD improvement and support the baPWV efficacy reported in our previous study (14).

Inflammation is involved in allergic symptoms. N-3 fatty acids possess anti-inflammatory properties, and animal studies have reported that administration of ALA alleviates symptoms of allergic conjunctivitis (26). ALA has reported anti-inflammatory effects (4–6). Additionally, ALA is metabolized in the body to become a precursor of eicosanoids, which are bioactive substances, and contributes to the regulation of inflammatory responses (25). Macrophage responses are deeply involved in allergic inflammation (54), and there are reports that ALA metabolites produced by gut bacteria suppress macrophage-associated inflammation (55). However, in this study, no reduction in ocular and nasal allergic symptoms was observed (Table 6). The participants in this study were individuals who reported cold sensitivity, and the presence or absence of allergic symptoms was not considered in participant selection. Among the subjects, 48.7% had ocular and nasal allergic symptoms at baseline (week 0) (data not shown), while more than half of the subjects had no ocular and nasal allergic symptoms. The small number of subjects with allergic symptoms may have reduced the study’s statistical power, potentially preventing adequate evaluation of the effect of ALA on ocular and nasal symptoms.

At week 12, serum ALA concentrations were significantly higher in both experimental oil groups compared to the control group (Table 8). In the experimental oil 2 group, the serum concentrations of EPA and DPA—metabolites of ALA—were also significantly higher at week 12 compared to the control group, while DHA showed a tendency toward higher levels. Furthermore, compared to the control group, the EPA:AA ratio was significantly higher in the experimental oil 2 group and showed a tendency toward higher levels in the experimental oil 1 group. The n-6:n-3 ratio was significantly lower in both experimental groups compared to the control group. The ingested ALA was transferred into the bloodstream, and in the group with higher intake, a portion was converted into EPA, DPA, and DHA. ALA is metabolized in the body and converted into longer-chain n-3 fatty acids, and the conversion rate is higher in premenopausal women than in men (56). Since this study targeted individuals who reported cold sensitivity, there were many female participants, with women under 50 years of age accounting for 42.7% of the total (data not shown). The intergroup observed not only in ALA but also in serum concentrations of EPA and DPA, the EPA:AA ratio, and the n-6:n-3 ratio may be related to the inclusion of many participants with high ALA conversion rates. In our previous study (14) of middle-aged and older women (45–64 years), 2.13 g ALA intake resulted in significantly higher serum ALA and EPA concentrations compared to the control group; however, no group differences were observed for DPA and DHA, which are longer-chain n-3 fatty acids. In contrast, in this study, which included approximately 40% premenopausal women with high n-3 fatty acid conversion rates, 2.03 g of ALA intake resulted in significantly higher serum ALA, EPA, and DPA levels, with DHA also showing a tendency toward higher levels. This finding is consistent with previous studies. Furthermore, a comparison of serum DHA concentrations before and after ALA intake showed that, although not statistically significant, the levels were lower after intake than before. However, this study could not clarify whether this decrease was caused by the consumption of DHA due to its role in cold sensitivity or vascular function, or whether it resulted from competition with other dietary fatty acids. It is known that some metabolites of EPA and DPA—produced in vivo through the metabolism of ALA—possess biological activity. DPA is metabolized by lipoxygenase to produce specialized pro-resolving mediators such as protectins and resolvins. These metabolites are known to prevent white blood cells from adhering to blood vessel walls and to alleviate vascular inflammation, and may be involved in the mechanism underlying the improvement in vascular function observed in this study (57). It has also been reported that ALA itself is metabolized to produce metabolites with anti-inflammatory effects (55). On the other hand, the blood fatty acid concentrations measured in this study showed large standard deviations. Compared with membrane lipidomics analysis, which is considered the gold standard for evaluating the bioavailability of food components (58), caution is warranted when interpreting the results. Furthermore, although the samples were treated under acidic conditions in this study, polyunsaturated fatty acids exhibit high reactivity at their double bonds, which may have influenced the measured concentrations (59).

This study has several limitations. First, since the study participants consisted of Japanese individuals, the effects of ALA intake in populations with different regions, lifestyles, and dietary habits remain unclear. Second, although AUC was calculated for skin blood flow and skin surface temperature, multiple comparisons were not controlled for. Third, participants were recruited from a subject registry and selection and exclusion criteria were applied to identify eligible subjects, but potential selection bias may exist. Fourth, while serum fatty acid concentrations were measured, their metabolites and fatty acids outside of serum were not measured. The effects of fatty acid metabolites and fatty acid uptake into membranes could not be directly evaluated. Furthermore, the serum fatty acid concentrations measured in this study exhibited large standard deviations, which may limit the interpretation of the results.

Despite these limitations, in a healthy Japanese population, ALA intake increased serum n-3 fatty acid concentrations and supported the recovery of skin blood flow and skin surface temperature following cold exposure. Furthermore, in individuals with decreased vascular endothelial function, ALA supported the improvement of FMD.

## Data Availability

The raw data supporting the conclusions of this article will be made available by the authors, without undue reservation.
